# Inhibition of granulocyte migration by tiotropium bromide

**DOI:** 10.1186/1465-9921-12-24

**Published:** 2011-02-27

**Authors:** Gabriela Vacca, Winfried J Randerath, Adrian Gillissen

**Affiliations:** 1 Robert-Koch-Hospital, St. George Medical Center, Leipzig, Germany; 2 Department for Pulmonary Medicine, Allergology, Sleep Medicine and Intensive Care, Hospital Bethanien, Universitaet Witten/Herdecke, Solingen, Germany; 3 Department of Pulmonary Medicine, General Hospital, Kassel, Germany

## Abstract

**Study objectives:**

Neutrophil influx into the airways is an important mechanism in the pathophysiology of the inflammatory process in the airways of patients with chronic obstructive pulmonary disease (COPD). Previously it was shown that anticholinergic drugs reduce the release of non-neuronal paracrine mediators, which modulate inflammation in the airways. On this basis, we investigated the ability of the long-acting anticholinergic tiotropium bromide to inhibit a) alveolar macrophage (AM)-mediated chemotaxis of neutrophils, and b) cellular release of reactive oxygen species (ROS).

**Method:**

AM and neutrophils were collected from 71 COPD patients. Nanomolar concentrations of tiotropium bromide were tested in AM cultured up to 20 h with LPS (1 μg/ml). AM supernatant was tested for TNFα, IL8, IL6, LTB4, GM-CSF, MIPα/β and ROS. It was further used in a 96-well chemotaxis chamber to stimulate the migration of fluorescence labelled neutrophils. Control stimulants consisted of acetylcholine (ACh), carbachol, muscarine or oxotremorine and in part PMA (phorbol myristate acetate, 0.1 μg/ml). Potential contribution of M_1-3_-receptors was ascertained by a) analysis of mRNA transcription by RT-PCR, and b) co-incubation with selective M-receptor inhibitors.

**Results:**

Supernatant from AM stimulated with LPS induced neutrophilic migration which could be reduced by tiotropium in a dose dependent manner: 22.1 ± 10.2 (3 nM), 26.5 ± 18,4 (30 nM), and 37.8 ± 24.0 (300 nM, p < 0.001 compared to non-LPS activated AM). Concomitantly TNFα release of stimulated AM dropped by 19.2 ± 7.2% of control (p = 0.001). Tiotropium bromide did not affect cellular IL8, IL6, LTB4, GM-CSF and MIPα/β release in this setting. Tiotropium (30 nM) reduced ROS release of LPS stimulated AM by 36.1 ± 15.2% (p = 0.002) and in carbachol stimulated AM by 46.2 ± 30.2 (p < 0.001). M3R gene expression dominated over M2R and M1R. Chemotaxis inhibitory effect of tiotropium bromide was mainly driven by M3R inhibition.

**Conclusion:**

Our data confirm that inhibiting muscarinic cholinergic receptors with tiotropium bromide reduces TNFα mediated chemotactic properties and ROS release of human AM, and thus may contribute to lessen cellular inflammation.

## Introduction

The pathogenesis of chronic obstructive pulmonary disease (COPD) is characterized by persistent neutrophilic inflammation of the airways and lung parenchyma [1,2]. They release cytokines, leukotrienes, reactive oxygen species (ROS), elastases and other proinflammatory mediators which correlate broadly with disease severity [3-5]. Neutrophils are attracted from the capillary bed into the airways through chemotactic mechanisms which are intensified during exacerbation [6,7]. With increasing disease severity activated mononuclear cells and monocytes/macrophages contribute more and more to the complex inflammatory process in the airways and in the bronchial submucosa of those patients [8].

Chemotaxis is a biological phenomenon whereby a cell type migrates through barriers (e.g. vessel walls, epithelial layers or tissue) toward the site of inflammation. These cells will initiate and maintain the inflammatory process through a variety of mechanisms including ROS release. In COPD, chemotaxis is not only regarded as an important pathologic feature of prolonged inflammation due to cigarette smoke inhalation, but may also be an appealing target for anti-inflammatory therapy. By reducing the neutrophil influx into the airways one should be able to reduce the burden of airway inflammation and, thus, change the natural history of the disease [9,10]. Unfortunately, inhaled corticosteroids have been shown to reduce neutrophilic inflammation in COPD patients just poorly at best [11-13]. Long-acting β2-agonists do not have intrinsic anti-inflammatory properties per se. In contrast, the long-acting muscarinic receptor antagonist tiotropium bromide has been shown a) to regulate release of chemotactic factors from human epithelial cells and macrophages in vitro [14], b) to inhibit airway remodelling and increase in smooth muscle mass in ovalbumin-sensitised guinea pigs [15,16], and c) to inhibit acetylcholine mediated proliferation of fibroblasts and myofibroblasts in vitro [17,18]. These observations may be based on induced mitogenesis by stimulated muscarinic receptors and mediator release, which can be lessened by muscarinic receptor antagonists [19].

We hypothesize that tiotropium may have potential antiinflammatory properties helping to explain its good clinical efficacy e.g. the reduction of exacerbation rate which can hardly be related to its bronchodilative function alone. Through reducing chemotaxis of neutrophils via inhibition pro-chemotactic properties of alveolar macrophages anticholinergic drugs and tiotropium in particular may impact the neutrophil and macrophage driven inflammation in COPD in a considerable way. This would be a new perspective how those compounds affect those patients. The rationale for this study was therefore to test tiotropium having anti-chemotatic properties in a macrophage and neutrophil containing cell system.

## Methods

In this study alveolar macrophages and neutrophils from COPD patients (n = 71) were used. They were recruited during out-patient or in-patient visits in our institution. Main inclusion criteria were a smoking history of ≥20 pack-years, COPD regardless of severity according GOLD -criteria (global initiative for lung disease [20]). Main exclusion criteria were an acute infection of the airways or the lung, other chronic lung diseases (except COPD), cancer or extra-pulmonary chronic diseases causing clinical instability. All patients gave written, informed consent. All of them had a clinical history, a physical examination and an X-ray from the chest prior to bronchoscopy and bronchoalveolar lavage (BAL). Appropriate investigations relating to the clinical presentation together with history including smoking history, spirometric and radiologic data as well as blood gas analysis were obtained from the records. The study was approved by the ethics committee of the Saxonian Chamber of Physicians, Dresden, Germany (approval No.: EK-BR-27/05-2).

Patients referred for bronchoscopy for various clinical reasons were invited to participate in the study. Prior to bronchoscopy short acting anticholinergic drugs (ipratropium bromide) were stopped for at least 12 h and the long acting tiotropium bromide for at least 48 h. Prednisolone was limited to 7,5 mg/day. Inhaled short- and long-acting β2-agonists, inhaled corticosteroids, further, antibiotics and theophylline were allowed.

BAL was performed according to standard procedure as recommended [21,22]. The bronchoscope was wedged in the right middle lobe or lingula and up to 140 ml of normal warmed (37°C) saline was instilled in 20-ml aliquots. Lavage fluid was immediately rinsed through gauze in order to remove surplus mucus. The fluid was centrifuged at 800 rpm for 10 minutes, 1 × 10^6^/ml isolated macrophages were plated on 24-well tissue culture plates in RPMI (Biochrom AG)/10%FCS/L-glutamine (2 mM, GIBCO/Invitrogen)/antibiotics (penicillin 100 IU/ml, streptomycin 100 μg/ml, amphotericin 0,25 mg/ml, Biochrom AG) and cultured up to 2 h at 37°C/5%CO2. After washing to remove unattached AM and other cells, cells were further cultured over night. For estimation of cell viability/cytotoxicity we used trypan blue and neutral red uptake assay as described elsewhere [23]. Viability had to be ≥90%, macrophage content of BAL cell differential ≥85%, and at least 5 million cells were all required for further analysis. Cell vitality and cellular purity (microscopic examination of 300 cells at magnification ×100) was always ≥95%. For all experiments a cellular concentration of 2 × 10^6 ^cells/ml was used. All experiments were run in triplicates.

Polymorphonuclear leukocytes were isolated from the blood of these patients according to standard procedure as described elsewhere [24]. They were labelled with Calcein AM (5 μg/ml, Molecular Probes) in a 30 min incubation step at 37°C/5% CO2 for later detection using a flourescence based detection system. Neutrophils were than washed twice with PBS, counted and resuspended in RPMI/FCS/L-glutamine/penicillin/strepto-mycin/amphotericin [25,26].

AM were washed and incubated at 37°C/5% CO_2 _for different time periods with RPMI/1%FCS/L-glutamine/penicillin/strepto-mycin/amphotericin with or without increasing concentrations of either acetylcholine (ACh: 1000; 100; 10; 1; 0.1 μM), carbachol, muscarin, oxotremorin (100, 10 and 1 μM) or LPS (10, 1 and 0.1 μg/ml; all reagents from Sigma Chemicals). In addition co-stimulation was done with LPS (0.1-1 μg/ml) and carbachol or muscarin (10-100 μM). After designated time periods, supernatants were strip off, centrifuged to remove remaining cells and cell detritus and frozen (-80°C) for cytokine quantification.

AM and neutrophils from these patients were prospectively distributed to the different assays. Due to large assay number and control experiments, as well as depending on the usefulness of the material and the cell number obtained from each patient, assays were run only with a certain proportion of patient samples: n = 20 for chemotaxis with tiotropium, n = 10 for LPS/carbachol/muscarin/tiotropium co-stimulation, n = 9 for controls with acetylcholin-esterase, n = 15 for RT-PCR experiments, n = 17 for ROS release experiments.

### Cytotoxicity

After AM-stimulation with LPS with or without tiotropium (3 nM, 30 nM, 300 nM) for 4 and 20 h, supernatants were stripped off. Remaining cells were washed three times with PBS and cytotoxicity was quantified. For the estimation of cell cytotoxicity we used neutral red uptake assay as described elsewhere [23]. Control cells (cultured with RPMI/1%FCS/antibiotics/glutamine) were set to 100% and used for normalisation. Proper staining with 2',7'-dihydrodichlorofluorescein diacetate (H_2_DCFDA) a fluorescent dye used in the cytotoxicity assay was confirmed by Fluorescence microscopy (Nikon).

### Chemotaxis

Cell migration of neutrophils was assayed using a 96-well Transwell chamber (pore diameter 3 μm, Corning). The bottom chamber was filled with culture supernatants generated from AM culture supernatant as described above. Isolated neutrophils (75 μl, 2 × 10^6 ^cells) were placed in the upper chamber. Spontaneous migration during incubation in 37°C/5% CO2 for 60 min. was determined using RPMI alone. RPMI/1%FCS/L-glutamine/penicillin/strepto-mycin/amphotericin in the bottom chamber functioned as positive control (= FCS induced migration). Migrated cells were quantified in a multi-well fluorescent plate reader (Fluostar, BMG), whereas the intensity of the calcein fluorescence signal detected at excitation 485 nm and emission 535 nm in the bottom chamber corresponds to neutrophil cells found there. The values of the spontaneous migration were set to 100% and used for normalisation of different experiments.

Different muscarinic receptor antagonists were tested to elucidate the role of specific M-receptors and their inhibitors on chemotactic activity in AM cultured in RPMI/FCS/L-glutamine/penicillin/streptomycin/amphotericin: telenzepine (M1R inhibitor, 0.01 μM), gallamine (M2R inhibitor, 100 μM), 4-DAMP (M3R inhibitor, 100 nM), tubocurarine (nicotinic receptor inhibitor, 100 μM), ipratropium (30 nM, Sigma Chemicals) and tiotropium (3 nM, 30 nM, 300 nM, provided by Boehringer Ingelheim).

### Cytokine measurements

Commercially available ELISA kits were used for the detection of human IL-8, IL6, TNF alfa, GM-CSF (IBL, Hamburg, Germany), LTB 4 (Cayman, USA), MIPα/β (Biosource Int., USA) in AM supernatants with minor modifications of the manufacturer's protocol.

### Reactive oxygen species (ROS)

Intracellular ROS formation was measured by the oxidant sensitive dye 2',7'-dichlorofluorescein diacetate (DCFH-DA [27]). The amount of fluorescence correlates with ROS released by the cells. AM were cultured under the above culture conditions (with or without the compounds to be tested), washed in PBS and then incubated with 1 μl DCFH-DA/1 ml PBS for 1 h at 37°C/5%CO2. Fluorescence was quantified with FLUOstar OPTIMA (BMG Labtech). Negative controls consisted of a) AM cultured without DCFH-DA, and b) AM preincubated with prednisolone (10 μM). LPS activated AM and PMA 0.1 μg/ml were used as positive controls.

### RT-PCR

For quantitative mRNA expression of muscarinic receptors of AM RT-PCR (Rotorgene 3000, Corbett Research) was used. Total RNA was isolated using Trizol (Invitrogen), quantified spectrophotometrically and 1 μg was reverse transcribed to produce cDNA (Superspcript III Platinum, Invitrogen). The cDNA was then used to determine gene expression levels of the muscarinic receptors M1R-M3 relative to β-actin. The PCR reaction was performed with primers described by Pieper et al. [17]. The specificity of PCR reactions was verified by melting curve analyses and electrophoresis (date not shown). The primers used had the following sequences: M1R for (5'-GGCACGCTGGCTTGTGA-3'), M1R rev (5'-TTCATGACGGAGGCATTGC-3'), M1R-Probe (FAM-5'-CTGGCCCTGGACTATGTGGCC-3-TAMRA'), M2R-for (5'- CCTGGAGCACAACAAAATCCA -3'), M2R-rev (5'- TCCCTGAACACAGTTTTCAGTCA-3'), M2R-Probe (FAM-5'-ATGGCAAAGCCCCCAGGGATCC-3-TAMRA'), M3R-for (5'-ACAGCCCCTCCGATGCA -3'), M3R-rev (5'-AACATTGTAGCTGCCGAAATGA -3'), M3R-Probe (FAM-5'-CTGCCCCCGGGAACCGTC -3-TAMRA'), β-actin for (5'-TGACGCCGGCTACAGCTT -3'), β-actin rev (5'-TCCTTAATGTCACGCACGATTT -3'), β-actin-Probe (FAM-5'-ACCACCACGGCCGAGCGG -3-TAMRA'). In order to exclude that our results were influenced by different muscarinic receptor expression due to varying culture conditions, RT-PCR was performed in AM-RNA a) without over night incubation (2 h after isolation from BAL), b) with overnight incubation in RPMI/10%FCS/L-glutamine/penicillin/streptomycin/amphotericin (for 20 h), c) in control cells (see above), and d) in AM stimulated with LPS (1 μg/ml) or e) carbachol (100 μM).

### Statistical analysis

Values were expressed as mean value ± SD (or ± SEM when indicated) of *n *experiments. For statistical analysis to compare the response of AM/neutrophils to tiotropium bromide with and without carbachol acetylcholine, muscarin and oxotremorin we used the Kruskall-Wallis test, the Mann-Whitney rank-sum test and the Wilcoxon signed-rank test respectably, if appropriate. Statistical significance was accepted at the level of p < 0.05. All statistical tests were performed using the SigmaStat software version (SPSS Science).

## Results

Patient characteristics are shown in table 1.

**Table 1:** Baseline parameters of patients (mean ± SD).

Parameter	Value
N	71
Women	n = 46 (64.8%)
Men	n = 25 (35.2%)
***Lung function and blood gas analysis***	

FEV1 (%predicted)	80.3 ± 23.1
FEV/FVC (%)	69.1 ± 7.6
pO2 (mmHg)	72.5 ± 12.1
pCO2 (mmHG)	37.0 ± 3.4
pH	7.4 ± 0.01
***Cell differential of bronchoalveolar lavage***	

Alveolar macrophage [%]	91.1 ± 7,2
Neutrophils [%]	5.2 ± 3,1
Lymphocyte [%]	1.9 ± 0,7
Eosinophils [%]	0.7 ± 0,4
Mast cells [%]	0.1 ± 0,3
Total cells [10^6^]	8.9 ± 5,5

### Chemotaxis

Supernatant from AM stimulated with LPS (0.1, 1 and 10 μg/ml) caused a concentration-dependent increase in the neutrophilic migration. A LPS concentration of 1 μg/ml resulted in an optimal chemotaxis, and was used in all consecutive experiments.

### Involvement of muscarinic receptors in LPS-mediated effects

**Figure 1:**
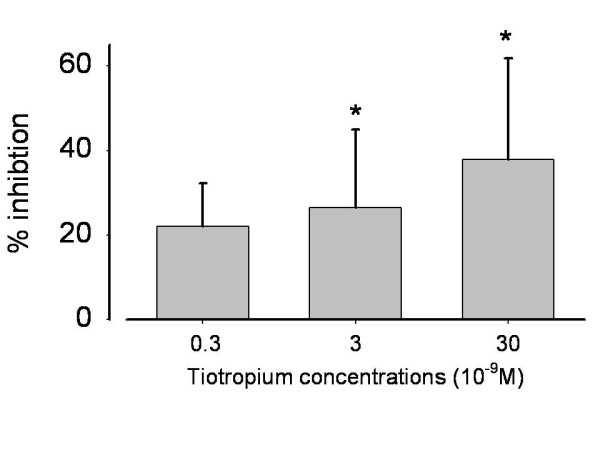
**Tiotropium inhibited chemotactic activity from LPS activated AM (test runs from left to right: n = 4, n = 9, n = 20 patients)**. Mean ± SD (standard deviation). * p < 0.001 compared with LPS activation without inhibition. Concentrations according to expected values in the airways after tiotropium inhalation http://www.rxlist.com/cgi/generic3/spiriva_cp.htm.

Supernatant from AM cultured with LPS and the anticholinergic drug tiotropium (30 nM) resulted in a significant (p < 0.001) reduction of neutrophil migration compared with supernatant from AM cultured with LPS alone (figure 1). Tiotropium had no effect on migration rates in the absence of LPS (data not shown). Taken together, these data suggest that activation of muscarinic receptors was involved in the LPS-mediated release of chemotactic factors. To further investigate this point, co-incubation experiments with AChE (acetylcholinesterase) added to LPS stimulated AM cell medium (n = 9) resulted in reduced migration rates of neutrophils: 154% ± 33% of control (LPS alone), 113% ± 17% (LPS+AChE, p value < 0.001), suggesting that acetylcholine release and consequent activation of muscarinic receptors are part of the signalling cascade activated by LPS.

To further explore this aspect, AM were stimulated with different muscarinic agonists, i.e. acetylcholine, carbachol, muscarin and oxotremorine. However, neither of these agonists induced neutrophilic migration alone, nor potentiated LPS-mediated effect [+45.5 ± 39.1% (LPS+ acetylcholine), +12.3 ± 12.7% (LPS+carbachol), +15.2 ± 19.8% (LPS+muscarin), +21.2 ± 17.1% (LPS+oxotremorine), +41.5 ± 23.2% (LPS)]. Taken together, these data suggest that ACH release and muscarinic receptor activation are a necessary component in LPS mediated effect, but not sufficient.

### Detection of which muscarinic receptor subtype is involved in LPS effects

**Figure 2:**
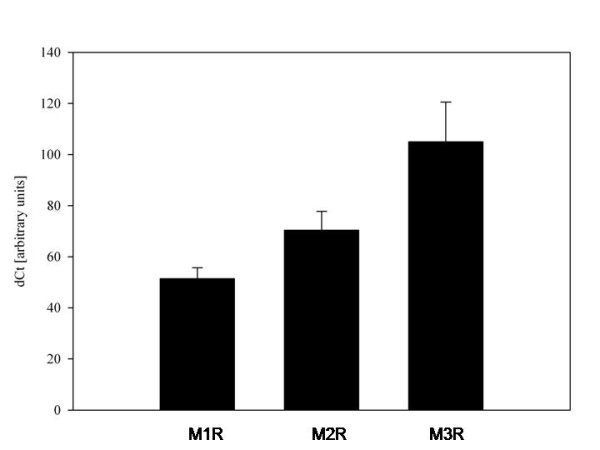
**Cellular mRNA levels of muscarinic M1R (MRC1), M2R (MRC2), M3R (MRC3) subreceptors in alveolar macrophages from COPD patients (n = 11)**. Mean+SD.

**Figure 3:**
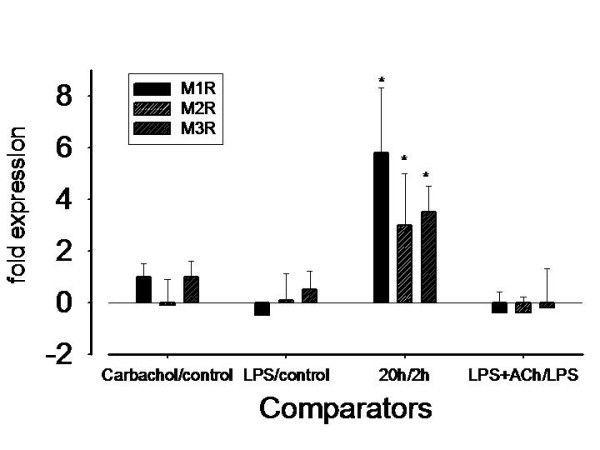
**Differences in muscarinic receptor (MRC1-3) expression profile in AM from n = 19 COPD patients depending on culture conditions**. X-axis represents comparisons always after an incubation time of 20 h: carbachol vs. control, LPS vs. control, 20 h vs. 2 h, LPS+ACh vs. LPS. Mean ± SD. * p = < 0.001 (repeated measures One Way ANOVA).

The analysis of muscarinic M1R, M2R and M3R mRNAs by RT-PCR showed that all the subtypes are present in AM, with muscarinic M3R mRNA transcripts dominating over M2R, and M1R (figure 2). 20 h after isolation from BAL, M-receptor expression increased compared with 2 h incubation (p < 0.001). At the 20 h time point M-receptor expressions did not vary regardless of stimulants added to the cell medium (figure 3).

**Figure 4:**
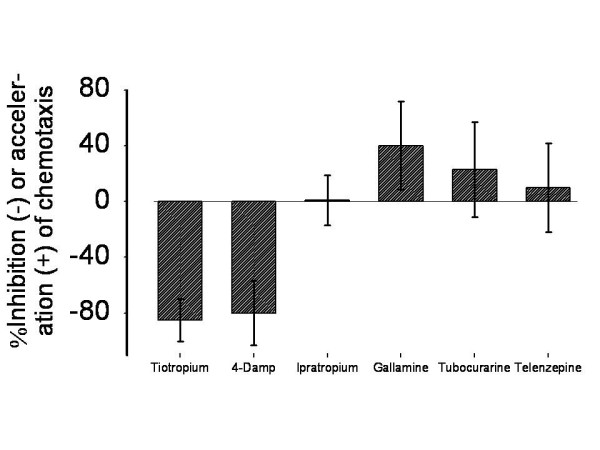
**Inhibition of AM induced chemotactic activity from tiotropium on neutrophils is predominantly driven by M3R-blockage (n = 10): Tiotropium 30 nM, 4-DAMP 100 nM (each: p < 0.01 vs. LPS stimulation alone), Ipratropium 30 nM, Gallamine 100 μM, Tubocurarine 100 μM, Telenzepine 10 nM**. Doses were adapted from earlier studies and customized to our assay conditions [43,63,64].

The muscarinic M3R antagonist 4-diphenylacetoxy-N-methylpiperidine methiodide (4-DAMP) and tiotropium (p < 0.01 vs. LPS stimulation alone) reduced neutrophilic migration rate in our chemotaxis system, whereas the muscarinic M1R-receptor antagonist pirenzepine and M2R antagonist gallamine did not (figure 4).

Taken together, these data suggest that, in this model, ACh exerts its activity through the activation of the human M3R, which is also the most expressed subtype in AM.

### Anticholinergic effects in reducing mediator release

**Figure 5:**
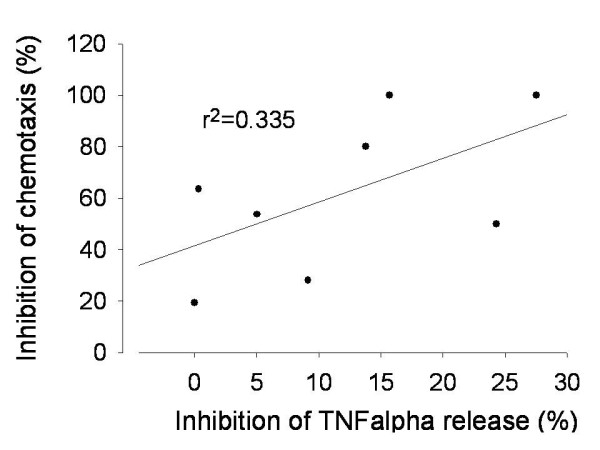
**Correlation of TNFα release of pre-cultured and LPS activated alveolar macrophages (AM) to the migration rate of neutrophils cultured with AM cell medium (Tiotropium 30 nM)**.

As expected, LPS exposure of AM resulted in an increase of many pro inflammatory mediators, as TNF-a, IL-8, IL-6, LTB4, GM-CSF and MIPα/β (data not shown). Coincubation with tiotropium (30 nM) resulted in a significant reduction of elevated TNFα secretion from LPS stimulated AM, which correlated fairly with the reduction of neutrophil migration rates (R^2^=0.335, p < 0.001, figure 5). Concerning the other cytokines, tiotropium had no significant effect (date not shown).

### Release of reactive oxygen species in AM

**Figure 6:**
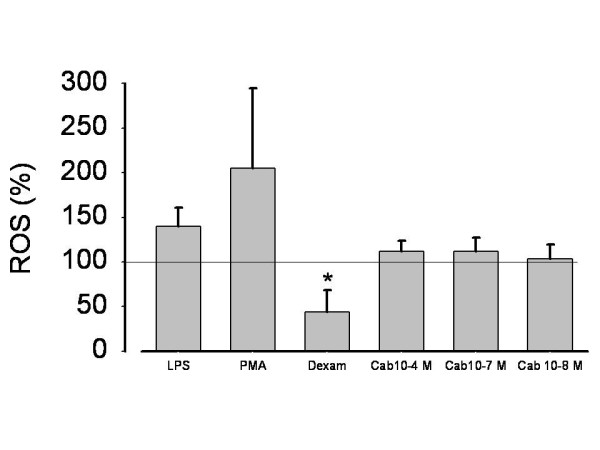
**ROS (reactive oxygen species) release from LPS, PMA or carbachol activated alveolar macrophages (AM) from 16 patients**. ROS production in unstimulated cells was defined as 100%, ROS in cell free medium was 0. LPS 1 μg/ml; Carb = carbachol in various concentrations; Dexam = Dexametasone 10^-7 ^M in LPS activated AM; PMA 0.1 μg/ml. Data expressed as mean ± SD. *p < 0.001 vs. LPS.

**Figure 7:**
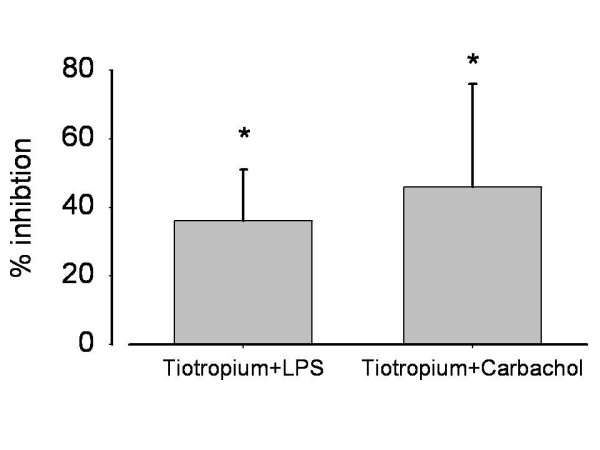
**Inhibition of ROS release by tiotropium (3 × 10^9 ^M) in LPS (1 μg/ml) or carbachol (10^-4 ^M) activated alveolar macrophages**. *p < 0.001, both comparisons with LPS or carbachol alone. Mean ± SD.

ROS are another important pro-inflammatory stimulus in COPD which is known to be a chemotactic factor. We therefore tested tiotropium bromide also to reduce ROS in order to further elucidate anti-inflammatory efficacy of this compound. Among the different stimuli tested, PMA (0.1 μg/ml) generated the highest ROS release in cultured AM, followed by LPS, which in turn is a stronger stimulus than carbachol. As expected, dexametasone (10 μM), which was used as positive control, substantially reduced LPS mediated ROS (figure 6). In 11 out of 16 patients tiotropium (30 nM) reduced ROS production from LPS stimulated AM by 36.1%. After stimulation with carbachol (100 μM), tiotropium induced a 46.2% reduction in ROS release; (p < 0.001 vs. LPS or carbachol alone; figure 7).

## Discussion

Clinical studies in COPD patients using inhaled anticholinergic tiotropium 18 μg once daily revealed reduced exacerbation rates as well as an improvement of lung function and the natural course of COPD [28,29]. The underlying mechanism how long-term treatment with tiotropium prevents exacerbations in these patients is unclear since the drug is regarded as a bronchodilator apparently lacking antiinflammatory capabilities in vivo [30,31]. COPD exacerbations are at the cellular level characterized by increasing systemic and bronchial inflammation which can be at least in part influenced by long-term treatment of inhaled corticosteroids [32-36]. It is unlikely that pure bronchodilation may cause this phenomenon because long-acting β2-agonist therapy alone is unable to reduce exacerbation rates and exacerbation severity to a clinically meaningful extent [37-39].

COPD has a high driving force to recruit inflammatory cells from the capillary bed into the airways [40] which is at least in part regulated by the muscarinic cholinergic system [41,42]. The ACh promoting effect on neutrophilic migration rate is dose dependent as Sato et al were able to demonstrate in ACh (1-100 μM) activated bovine alveolar macrophages [43]. In concordance with other studies, our data demonstrate a) that ACh drives chemotaxis since AChE reduces LPS-induced neutrophilc migration rates by about 40%, and b) that long-acting muscarinic receptor antagonist tiotropium bromide has additional anti-inflammatory capabilities due to reducing chemotactic activity of cultured AM in vitro. In regard to chemotaxis induction, ACh proved to be a much weaker driving force than LPS, suggesting that its role in LPS-mediated effects is necessary, but not sufficient to induce migration. Also, exogenously added ACh did not further enhance LPS efficacy, suggesting that LPS is per se already very efficacious and maximal chemotaxis was already reached (ceiling effect). Because of that, we run our experiments solely with LPS. In our experimental setup tiotropium bromide reduced LPS-mediated chemotaxis, possibly through an antiinflammatory effect as indicated by the reduction of TNFα release. Anti-inflammatory drugs, such as phosphodiesterase 4 inhibitors, have been shown to reduce TNFα and chemotaxis in sputum of COPD patients [44] which is at least in part mediated by mitogen activated protein (MAP) kinase pathway [45]. Our data may point to a similar effect of tiotropium bromide. However, TNFα reduction correlated weakly with the drop of chemotaxis, hinting that other mechanisms may be involved in tiotropium bromide influence in human alveolar macrophage/neutrophil chemotaxis. Because the supernatant used in the chemotaxis chamber contained tiotropium it might have exerted an additional effect on neutrophils which also express muscarinic receptors [14].

Surprisingly, tiotropium bromide failed to have any impact on the chemotactic mediators IL8, IL6, LTB4 and GM-CSF but not on cellular ROS release. Various other studies in myocytes also emphasize the stimulating effect of ACh on ROS production which is caused by activation of PI3K, Src-kinases or the ERK pathway at last leading to opening of mitochondrial K^ATP ^channels and ROS release [46,47]. ROS by themselves trigger chemokine production in inflammatory cells thus enhancing the inflammatory process [48,49]. The involvement of the cholinergic system in ROS and ROS-mediated cytotoxicity make it an ideal system to test muscarinic receptor inhibitors for their cell protective function. In this context we were able to demonstrate that nanomolar concentrations of tiotropium protect cells by reducing the oxidant load produced by alveolar macrophages and LPS-mediated cytotoxicity. The concentration needed to obtain the cell protective effect in our cytotoxicity assay is in a physiologic range which can be achieved in human airways after inhalation. However, best protection was seen at high concentrations (300 nmolar). Possibly, reduction of ROS release is another mechanism by which tiotropium bromide limits chemotaxis. Our data are in good concordance with Wollin and Pieper describing a dose-dependent reduction of pulmonary neutrophilic inflammation of inhaled tiotropium (0.01 - 0.3 mg/ml) in smoke exposed mice [50], but they seem to defy Perng et al. [31], who didn't detect any antiinflammatory effect of tiotropium in humans. The difference between the three studies may be due to the lack of a placebo arm in the COPD trial in which all 3 treatment arms (fluticasone/sameterol, fluticasone/tiotropium, tiotropium alone) failed to show differences in the cellular component in sputum although differences in some inflammatory mediators were observed.

Profita et al. (2008) demonstrated in 16HBE cells that acetylcholine-mediated IL-8 release significantly increased chemotaxis of neutrophils. This effect was inhibited by tiotropium bromide [51]. In the A549 and MonoMac6 cell line as well as in alveolar macrophages derived from non-smoking patients without a pulmonary disease tiotropium bromide decreased the release of chemotactic mediators after stimulation with high doses of ACh (100 μM) by about 70% [14]. Because ACh did not influence cellular release of IL-8 and monocyte chemotactic protein-1 (MCP-1) the authors suggested that leukotriene B4 (LTB4) was the driving force of chemotactic activity.

However, the authors failed to clearly demonstrate that the reducing effects of tiotropium bromide correlate with inhibition of cellular LTB4 release. Nevertheless there is clear relationship between ACh mediated LTB4 release and chemotaxis of inflammatory cells in COPD patients, who have higher LTB4 amounts in induced sputum, which correlates with number of sputum neutrophils, than healthy volunteers. ACh (100 μM) stimulated sputum cells from COPD patients but not from non-smokers release LTB4. Also in blood monocytes LTB4-production was ACh sensitive. These effects could be blocked by an inhibitor of extra cellular signal-regulated kinase, but also by the anticholinergic compound oxitropium bromide at a concentration of 10 μM [52]. However, this drug concentration is high when taking into account that by inhalation only nanomolar concentrations can be achieved locally http://www.rxlist.com/cgi/generic3/spiriva_cp.htm. The reason why we failed to demonstrate an inhibitory effect of tiotropium bromide on IL8 or LTB4 release from AM may be dependent on the chosen cells, since we evaluated human AM and human neutrophils instead of using a cell line. Because of working with primary cells, we think that our model is more representative of the situation in the airways of COPD patients.

It seems reasonable that M-receptor blockage inhibits chemotaxis, reduces anti-proliferative effects and ROS release, since activation of the cholinergic system has been shown a) to induce proliferative processes in airway tissue in vitro [53] as well as in vivo [16,19], b) to induce eosinophilic chemotactic activity [43,54], and c) ACh potentates the release of pro-inflammatory 15-HETE and prostaglandin E2 [55]. Further, cell proliferation of fibroblasts and myofibroblasts could be blocked by the nicotinic antagonist *n*-tubocurarine and antimuscarinic compounds [17,53], and progression of airway smooth muscle remodeling in a allergen challenge asthma model could be minimized using tiotropium bromide [16].

M1R, M2R and M3R mRNAs were all present in AM. M3R mRNA transcripts dominate over M2R, and M1R and expression increased 20 h compared with the beginning. We could confirm previous studies also showing muscarinic receptor expression in cell cultures. Differences in the extent of mRNA transcript levels are most likely related to different cell types [51] or different experimental set up and patient characteristics [52].

Our study has some shortcomings. First, although 71 patients were recruited, the number of single measurements of each assay block was low, which was due to the numerous controls and limited cell number per patient. However, due to strict selection criteria, data from the different experimental settings still are comparable. Second, due to numerous controls, number of single measurements seems low, and would have otherwise resulted in better statistics. This relates in particular to the weak TNFα/chemotaxis correlation. Third, in only about 50% of the cytotoxicity and ROS experiments in which the various inhibitors were tested, tiotropium bromide revealed inhibitory efficacy. This observation is not unique for our work since it has been found also in other studies on this subject. Blaas et al stimulated primary epithelial lung cells with carbachol (100 μg/ml) and found only in 5 out of 12 patients enhanced IL-8 release. In their experiments neutrophilic migration could be induced by carbachol in about 40% of their patients [56]. Subject variability [57,58], health status, varying nicotine consumption [59], cell type [60] and species differences [61,62] may be confounding factors contributing to this phenomenon. Forth, the number of migrated cells in our chemotaxis chamber system seems low in comparison to other publications. To detect more objectively migrated neutrophils and in order to enable high output measurements we used a fairly new chemotaxis chamber detecting fluorescence as the cellular marker for leukocyte migration. Other papers simply count the cells by light microscopy in the adjacent chamber as well as the filter whereas our system only detects truly migrated cells disregarding those cells sticking in the filter. Our assay has been described to comprise significant advances quantifying leukocyte chemotaxis and explains the differences to previous publications [26].

## Conclusion

Our results show that tiotropium bromide inhibits AM mediated chemotaxis of neutrophils. This effect correlates at least in part by concomitant TNFα reduction. Further, it reduces cellular proinflammatory activities such as the generation of ROS. Experiments with selective M -receptor inhibitors indicate that the M3R subtype is responsible for tiotropium bromide inhibition of chemotaxis.

## Competing interests

GV declares that she has no competing interests. WG and AG have received consulting fees, speaking fees, and grant support from Boehringer Ingelheim Pharma Germany.

## Authors' contributions

All authors have made substantial contributions. GV established the assays, and coordinated the work in the laboratory. She carried out the molecular genetic studies as well the immunoassays GV wrote the first version of the manuscript. WR helped in revising the manuscript critically for important intellectual content. AG planed the conception and design of the study and applied for the funding. He recruited the patients, collected the clinical data, wrote the final version of the manuscript and gave final approval of the version to be published. All authors read and approved the final manuscript.

## Funding

This study was funded by Boehringer Ingelheim GmbH, Germany
